# Dynamin 1-mediated endocytic recycling of glycosylated N-cadherin sustains the plastic mesenchymal state to promote ovarian cancer metastasis

**DOI:** 10.1093/procel/pwaf019

**Published:** 2025-04-09

**Authors:** Yuee Cai, Zhangyan Guan, Yin Tong, Weiyang Zhao, Jiangwen Zhang, Ling Peng, Philip P C Ip, Sally K Y To, Alice S T Wong

**Affiliations:** School of Biological Sciences, University of Hong Kong, Pokfulam Road, Hong Kong SAR, China; School of Biological Sciences, University of Hong Kong, Pokfulam Road, Hong Kong SAR, China; Department of Pathology, School of Clinical Medicine, University of Hong Kong, Queen Mary Hospital, Pokfulam Road, Hong Kong SAR, China; School of Biological Sciences, University of Hong Kong, Pokfulam Road, Hong Kong SAR, China; School of Biological Sciences, University of Hong Kong, Pokfulam Road, Hong Kong SAR, China; Aix-Marseille Université, Centre Interdisciplinaire de Nanoscience de Marseille, Marseille, 13288, France; Department of Pathology, School of Clinical Medicine, University of Hong Kong, Queen Mary Hospital, Pokfulam Road, Hong Kong SAR, China; School of Biological Sciences, University of Hong Kong, Pokfulam Road, Hong Kong SAR, China; Laboratory for Synthetic Chemistry and Chemical Biology Limited, Hong Kong Science and Technology Parks, Hong Kong SAR, China; School of Biological Sciences, University of Hong Kong, Pokfulam Road, Hong Kong SAR, China

**Dear Editor,**

Ovarian cancer is the leading cause of death among gynecologic malignancies, primarily due to metastatic disease, where current therapies are largely ineffective (5-year survival rate < 25%). Epithelial-to-mesenchymal transition (EMT) is a critical process that confers metastatic plasticity to ovarian cancer cells, enabling aggressive peritoneal dissemination, and contributing to poor clinical outcomes. EMT enhances anoikis resistance, allowing ovarian cancer spheroids to survive in ascitic fluid (Loret et al., 2019). These mesenchymal spheroids effectively penetrate the mesothelial lining and implant at secondary sites. To colonize, these cells undergo mesenchymal-to-epithelial transition to regain proliferative capacity. EMT is predominantly regulated by transcription factors such as snail family transcriptional repressor 1 (*SNAI1*), twist family BHLH transcription factor 1 (*TWIST1*), and zinc finger E-box binding homeobox 1 (*ZEB1*), which poses challenges for direct inhibition (Nieto et al., 2016). Cadherin switching, particularly the upregulation of N-cadherin associated with enhanced metastatic behaviors, is a key feature of EMT (Mrozik et al., 2018). However, the underlying mechanisms driving this process remain unclear.

To discover pharmacologically exploitable EMT drivers in ovarian cancer, we used a robust master regulator (MR) algorithm (Ru et al., 2019) to prioritize EMT regulators from over 8,000 patient samples across 20 cancer types in The Cancer Genome Atlas Program (TCGA) (Fig. S1A). By employing hallmark epithelial and mesenchymal signatures, we differentiated patient subtypes and compared differentially regulated genes. Using regulons inferred by the Algorithm for the Reconstruction of Accurate Cellular Networks (ARACNE), we identified approximately 6,700 activated and 9,000 repressed EMT MRs, including known transcription factors like *SNAI1*, *SNAI2*, *ZEB1*, *ZEB2*, and *TWIST1*, validating our approach (data not shown). Further screening for non-transcription factors led us to discover dynamin 1 (*DNM1*) as a novel MR (Fig. 1A and 1B). In TCGA ovarian cancer samples, *DNM1* expression negatively correlated with E-cadherin and positively correlated with N-cadherin (Fig. S1B). Elevated *DNM1* levels were observed in the mesenchymal molecular subtype compared to other subtypes, and in advanced-stage patients compared to those in early stages (Fig. 1C and 1D). Higher *DNM1* levels in ovarian tumor tissue were linked to poorer progression-free and post-progression survival (Fig. S1C and S1D). In contrast, *DNM2* showed no difference, while *DNM3* exhibited lower expression in tumors (Fig. S1C). Moreover, we confirmed higher DNM1 expression in intermediate and mesenchymal ovarian cancer cell lines compared to epithelial lines (Fig. 1E). These findings underscore a significant association between DNM1 and EMT in ovarian cancer.

**Figure 1.**
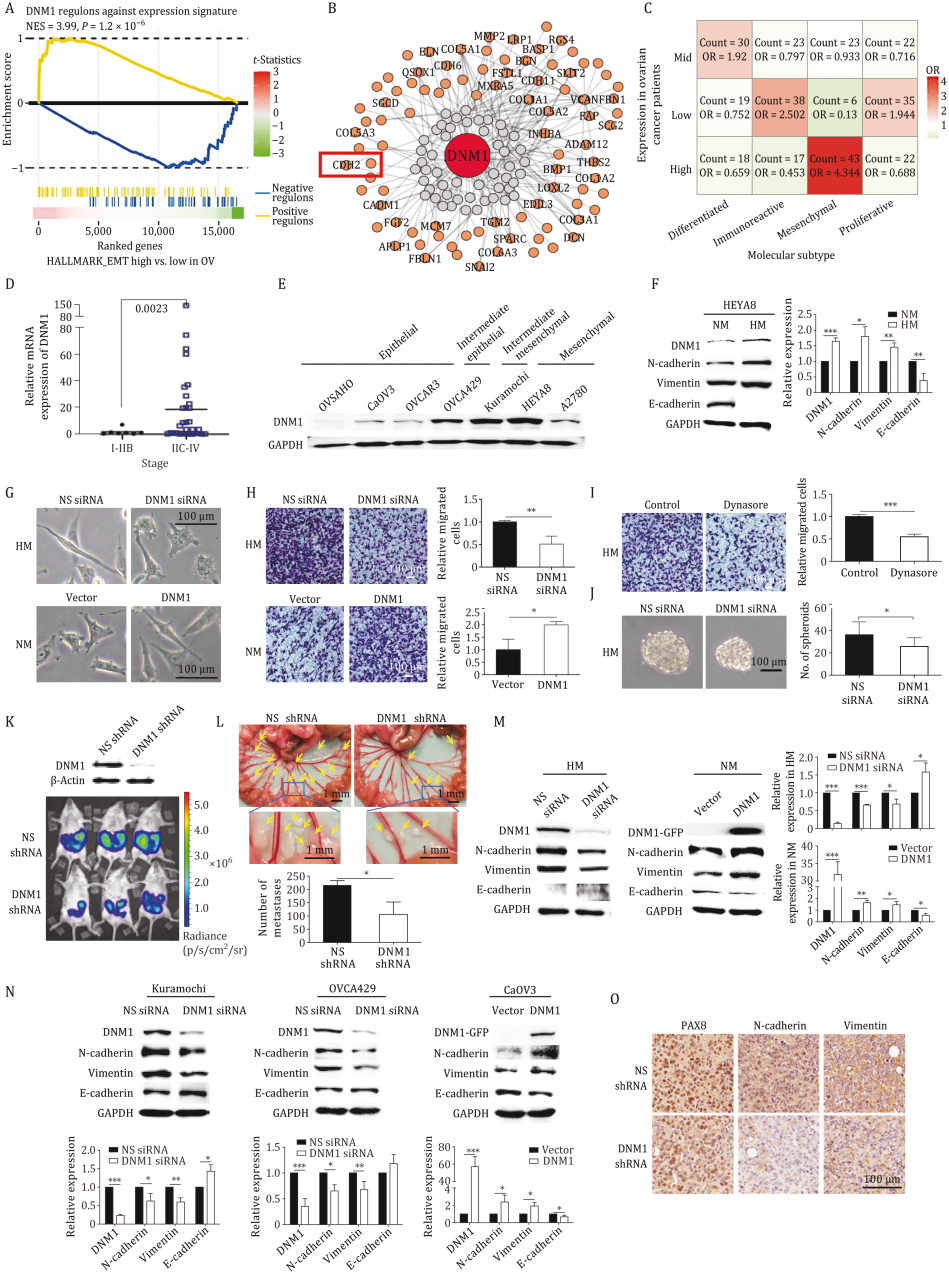
**DNM1 is a MR of EMT in ovarian cancer and promotes metastasis.** (A) GSEA analysis revealed a significant correlation between DNM1 expression and the HALLMARK_EPITHELIAL_MESENCHYMAL_TRANSITION gene set. (B) A gene regulatory network was constructed to illustrate the relationship between DNM1 and its associated regulons from the HALLMARK_EPITHELIAL_MESENCHYMAL_TRANSITION using ARACNE. (C) The odds ratio of high, mid, or low DNM1 expression levels across different molecular subtypes of ovarian cancer was calculated. (D) Quantitative real-time PCR (qPCR) analysis was performed on DNM1 expression in high-grade serous ovarian cancer patient samples at early (Stage I–IIB; *n* = 7) and late (Stage IIC–IV; *n* = 31) stages (unpaired *t*-test). (E) DNM1 expression in ovarian cancer cells with varying EMT phenotypes was analyzed via Western blot, with GAPDH serving as a loading control. (F) Protein expression levels of DNM1, N-cadherin, vimentin, and E-cadherin were assessed in NM and HM cells. (G) Morphologies of HM cells transfected with nonspecific (NS) or DNM1 siRNA (upper panel) and NM cells transfected with either a control vector or DNM1 overexpression vector (lower panel) were observed under a light microscope. (H) Migration assays were conducted on HM cells transfected with NS or DNM1 siRNA (upper panel) and NM cells transfected with either a control vector or DNM1 overexpression vector. Migrated cells were stained with crystal violet and counted in five random fields of view. (I) HM cells treated with vehicle control or dynasore (100 µmol/L) underwent migration assays. Migrated cells were stained with crystal violet and counted in five random fields of view. (J) Sphere formation assays were performed on HM cells transfected with NS or DNM1 siRNA. Representative spheroids are shown, and the number of spheres was counted. (K) DNM1 expression was evaluated in HM cells stably transfected with NS or DNM1 shRNA (upper panel). These cells were intraperitoneally injected into NOD/SCID mice (*n* = 3 per group, repeated twice). Peritoneal metastasis was visualized using *in vivo* bioluminescence imaging. (L) Metastatic nodules (indicated by arrows) on the mesenteries were counted after the mice were sacrificed. (M) Protein expression of DNM1, N-cadherin, vimentin, and E-cadherin was analyzed in HM cells transfected with NS or DNM1 siRNA, and NM cells transfected with either a control vector or DNM1 overexpression vector. (N) Protein expression of DNM1, N-cadherin, vimentin, and E-cadherin was evaluated in Kuramochi cells transfected with NS or DNM1 siRNA, OVCA429 cells transfected with NS or DNM1 siRNA, and CaOV3 cells transfected with either a control vector or DNM1 overexpression vector. (O) Immunohistochemistry was performed to assess PAX8 (a tumor marker), N-cadherin, and vimentin in metastatic tumors formed by HM cells with NS or DNM1 shRNA. GAPDH served as a loading control for all Western blot analyses. Band intensities were quantified using ImageJ. Data represent three independent experiments and are presented as mean ± standard deviation (SD). *P < 0.05; **P < 0.01; ***P < 0.005.

To investigate the functional role of DNM1, we utilized an isogenic ovarian cancer model derived from the HEYA8 cell line, where highly metastatic (HM) cells exhibited increased migration and peritoneal metastasis compared to non-metastatic (NM) cells (To et al., 2017). HM cells displayed elevated DNM1 expression, with higher N-cadherin and vimentin levels and reduced E-cadherin expression (Fig. 1F). DNM1 knockdown significantly inhibited mesenchymal morphology and migration (Fig. 1G and 1H), but this reduction in migration was rescued by the EMT inducer transforming growth factor beta 1 (TGFβ1) (Fig. S2A). Knockdown was confirmed via Western blot (Fig. S2B). Conversely, overexpressing DNM1 in NM cells induced EMT features, including spindle-like morphology and enhanced migration (Fig. 1G and 1H). DNM1 did not affect cell growth in either condition (Fig. S2C). Treatment with dynasore, a dynamin inhibitor, significantly reduced the migration of HM cells (Fig. 1I). In sphere formation assays, DNM1 knockdown decreased both spheroid size and number (Fig. 1J). To evaluate the *in vivo* role of DNM1, luciferase-expressing HM cells stably transfected with either nonspecific (NS) or DNM1-specific shRNA were injected into female non-obese diabetic/severe combined immunodeficient (NOD/SCID) mice. DNM1 shRNA cells showed less peritoneal dissemination and fewer metastatic tumors than NS shRNA controls (Fig. 1K and 1L), indicating that DNM1 promotes metastatic colonization.

Next, we examined the impact of DNM1 on EMT markers E-cadherin, N-cadherin, and vimentin. Silencing DNM1 markedly decreased N-cadherin and vimentin levels in HM, Kuramochi, and OVCA429 cells, while DNM1 overexpression increased these markers in NM and CaOV3 cells; however, E-cadherin levels showed variable effects (*P-*values ranging from 0.0117 to 0.144) (Fig. 1M and 1N). Given that N-cadherin is a regulon of DNM1 (Fig. 1B), we hypothesize that DNM1 regulates EMT through N-cadherin. Supporting this, DNM1 shRNA resulted in reduced N-cadherin levels compared to the NS control in mouse xenografts (Fig. 1O).

DNM1 is essential for vesicle scission during endocytosis and vesicular trafficking, and is predominantly expressed in neurons to facilitate rapid endocytosis and recycling of synaptic vesicles. In cancer, DNM1 has been shown to regulate the endocytosis of TNF-related apoptosis-inducing ligand (TRAIL) death receptors, thereby inhibiting apoptosis, while its activation through the protein kinase B (Akt)/glycogen synthase kinase-3 beta (GSK3β) pathway may disrupt clathrin-mediated endocytosis, promoting epidermal growth factor receptor (EGFR) signaling, and hence proliferation (Meng, 2017). To assess its effect on N-cadherin turnover, we conducted cell surface biotinylation and internalization assays (Fig. 2A). HM cells, with higher DNM1 expression, showed significantly increased N-cadherin endocytosis (Fig. 2B), which was inhibited by DNM1 silencing (Fig. 2C). Cadherin turnover is mainly regulated by clathrin- and caveolae-mediated endocytosis. Knockdown of caveolin-1, but not clathrin, inhibited N-cadherin endocytosis (Fig. S2D and S2E), indicating that DNM1 mediates N-cadherin endocytosis through a caveolae-dependent pathway. Further biotinylation and recycling assays (Fig. 2D) demonstrated that DNM1 silencing impeded N-cadherin recycling in HM cells, while overexpression in NM cells enhanced it (Fig. 2E and 2F). Treatment with the proteasome inhibitor MG132 prevented N-cadherin reduction after DNM1 knockdown, suggesting DNM1 could divert N-cadherin from degradation to recycling (Fig. S2F). Decreased colocalization of DNM1 with Rab11, a recycling endosome marker, was observed in HM cells treated with DNM1 siRNA, while NM cells overexpressing DNM1 showed increased colocalization (Figs. 2G and S3A). To evaluate the effects of DNM1 on migration, we performed scrape wound assays and Golgi tracking, revealing that DNM1 depletion impaired Golgi positioning and the directional recycling of N-cadherin-containing vesicles in HM and Kuramochi cells (Figs. 2H, 2I, S3B and S3C). Furthermore, treatment with primaquine, an endosomal recycling inhibitor, decreased N-cadherin levels and recycling (Figs. 2J, S3D and S3E) and impaired migration and Golgi polarization (Figs. 2K, 2L, S3F and S3G). No noticeable directional changes were observed in E-cadherin under these treatments (Fig. S3H and S3I), further supporting the specific role of DNM1 in N-cadherin endocytosis and recycling.

**Figure 2.**
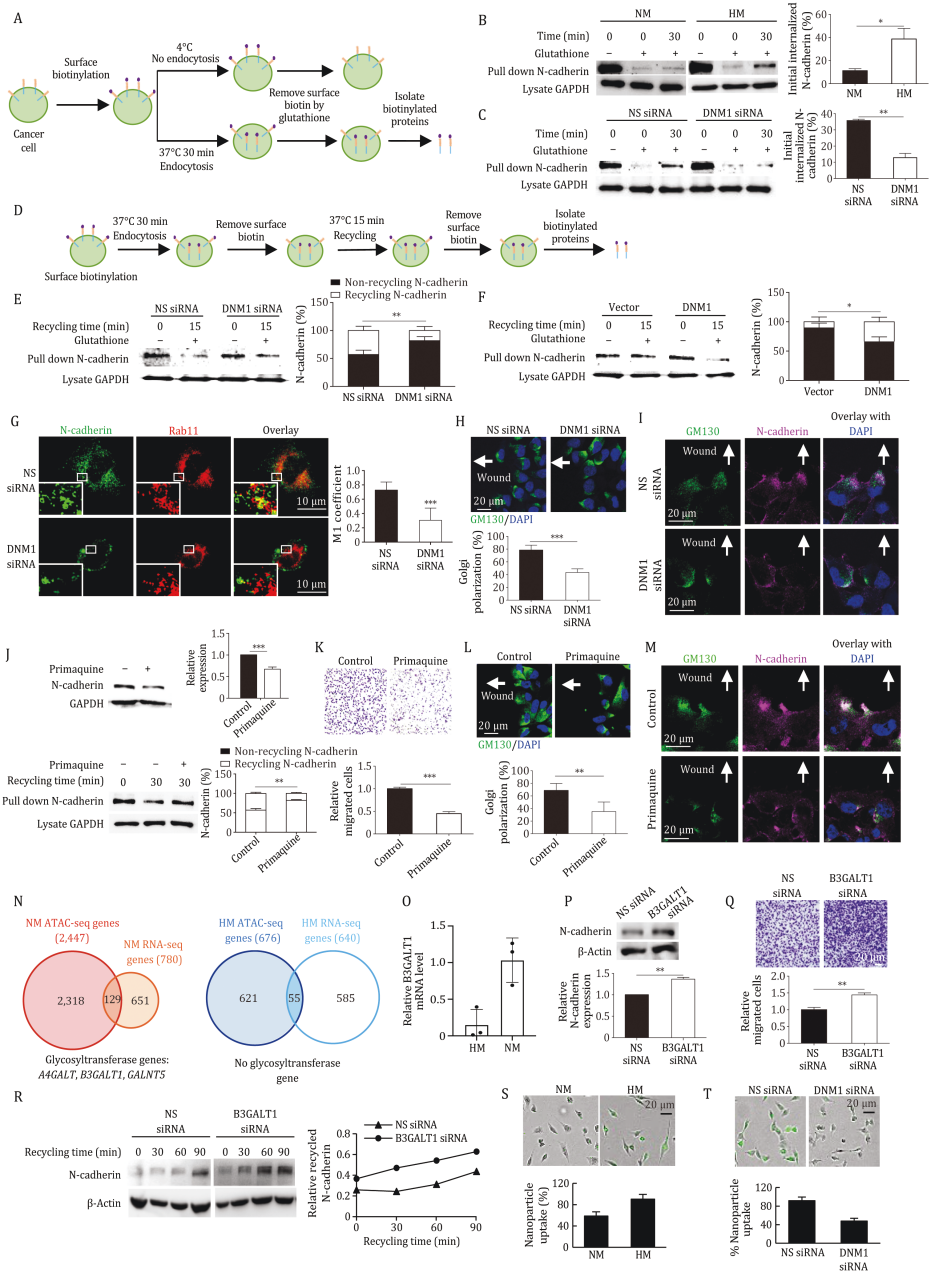
**DNM1 enhances the endocytic recycling of N-cadherin**. (A) A simplified workflow of the endocytosis assay using biotin labeling is shown. Surface proteins were biotinylated and incubated at 4°C (control) or 37°C for endocytosis. Surface biotin was removed with glutathione, followed by cell lysis to isolate biotinylated proteins using avidin. (B) The endocytosis of N-cadherin was evaluated in NM and HM cells using the biotin labeling assay and Western blot analysis. (C) The endocytosis of N-cadherin was evaluated in HM cells transfected with either nonspecific (NS) or DNM1 siRNA, using the biotin labeling assay and Western blot. (D) A simplified workflow of the recycling assay using biotin labeling is shown. Surface proteins were biotinylated and incubated at 37°C for endocytosis. After removing surface biotin with glutathione, cells were incubated again at 37°C to allow for recycling. Surface biotin was removed once more, followed by cell lysis to isolate non-recycled biotinylated proteins using avidin. (E) Recycling of N-cadherin was compared in HM cells transfected with NS or DNM1 siRNA using the biotin labeling assay and Western blot. N-cadherin at recycling time 15 min indicates non-recycled N-cadherin, as the biotin moieties of recycled N-cadherin returning to the cell membrane have been removed. The recycled N-cadherin was calculated as the total biotinylated N-cadherin endocytosed into the cytoplasm minus the non-recycled N-cadherin. (F) Recycling of N-cadherin was also assessed in NM cells transfected with either a control or DNM1 overexpression vector, using the biotin labeling assay followed by Western blot. (G) HM cells transfected with NS or DNM1 siRNA were stained for N-cadherin and Rab11 (a marker of recycling endosomes). Colocalization was observed via confocal imaging. Colocalization coefficient M1 was analyzed to describe the amounts of green pixels colocalizing with red pixels relative to the total green pixels. (H) Cell-free gaps were created in HM monolayers following transfection with NS or DNM1 siRNA. Golgi orientation was visualized using the Golgi marker GM130, and the number of cells exhibiting correct polarity was counted. (I) The indicated cells were co-stained with GM130 and N-cadherin and observed by confocal imaging. Cell nuclei were counterstained with DAPI. Arrows indicate the expected Golgi direction toward the wound. (J) HM cells were treated with either vehicle control or primaquine (100 μmol/L), a recycling inhibitor. N-cadherin expression was assessed by Western blot (upper panel). A biotin recycling assay was also performed, followed by Western blot for N-cadherin (lower panel). (K) Migration assays were conducted on HM cells treated with either vehicle control or primaquine. Migrated cells were stained with crystal violet and counted in five random fields of view. (L) Cell-free gaps were created in the HM monolayer treated with either vehicle control or primaquine. Golgi orientation was visualized by staining with the Golgi marker GM130, and the number of cells with correct polarity was counted. (M) The indicated cells were co-stained with GM130 and N-cadherin and observed by confocal imaging. Nuclei were counterstained with DAPI. Arrows indicate the expected Golgi direction toward the wound. (N) ATAC-seq and RNA-seq analyses were performed on NM and HM cells (three replicates each). Differentially accessible peak-associated genes were overlapped with upregulated genes in NM or HM cells. (O) Higher expression of B3GALT1 in NM cells compared to HM cells was confirmed by qPCR, using actin as a loading control. (P) N-cadherin expression was assessed by Western blot in NM cells transfected with NS or B3GALT1 siRNA. (Q) Migration assays were performed following the transfection of NM cells with NS or B3GALT1 siRNA. Migrated cells were stained with crystal violet and counted in five random fields of view. (R) NM cells were transfected with NS or B3GALT1 siRNA. After calcium chelation to induce N-cadherin internalization, cell surface proteins were allowed to recover for indicated time points. Cell surface proteins were biotinylated, and recycled N-cadherin was analyzed by Western blot. (S) NM and HM cells were treated with a dendrimer carrying fluorescein-labeled siRNA (20 nmol/L; green). (T) HM cells were transfected with NS or DNM1 siRNA, followed by treatment with the dendrimer carrying fluorescein-labeled siRNA (20 nmol/L; green). The percentage of cells that internalized the dendriplexes was quantified by counting the number of fluorescent signal-positive cells, normalized to the total cell number in the field. Band intensities were quantified using ImageJ. Data represent three independent experiments and are presented as mean ± SD. *P < 0.05; **P < 0.01; ***P < 0.005.

N-glycosylation is a crucial protein modification that regulates cadherin function (Carvalho et al., 2016). By integrating assay for transposase-accessible chromatin with sequencing (ATAC-seq) and RNA-seq (Fig. S4A and S4B), we identified three glycosyltransferase genes— lactosylceramide 4-alpha-galactosyltransferase (*A4GALT*), beta-1,3-galactosyltransferase 1 (*B3GALT1*), and polypeptide N-Acetylgalactosaminyltransferase 5 (*GALNT5*)—with increased chromatin accessibility and expression in NM compared to HM cells (Fig. 2N and 2O). While A4GALT and GALNT5 have been linked to EMT, the role of B3GALT1 remains unclear. Knockdown of B3GALT1, confirmed by qPCR (Fig. S4C), led to elevated N-cadherin levels and enhanced migration in both NM and Kuramochi cells (Figs. 2P, 2Q, S4D and S4E). Endocytosed cadherins can either be recycled or degraded in the lysosomes. To investigate whether B3GALT1 influences the lysosomal degradation of N-cadherin, we used ammonium chloride (NH_4_Cl), a lysosome inhibitor that increases intralysosomal pH and alters endosome-lysosome fusion rates. NH_4_Cl treatment restored N-cadherin levels, indicating ongoing lysosomal degradation in NM cells (Fig. S4F). B3GALT1 knockdown further increased N-cadherin protein levels in lysosome-inhibited cells, indicating that B3GALT1 reduces N-cadherin stability independently of lysosomal degradation (Fig. S4F). It also accelerated N-cadherin recycling in NM cells (Fig. 2R). Together, these findings suggest that B3GALT1 could inhibit N-cadherin recycling and suppress EMT.

We previously developed self-assembling supramolecular dendrimers for drug delivery (Ma et al., 2024). These nanoparticles utilize various endocytic pathways for cellular entry, particularly caveolae-dependent endocytosis, which minimizes lysosomal degradation and enhances cargo release (Blanco et al., 2015). Next, we investigated the therapeutic potential of DNM1-mediated endocytosis in our dendrimer-based system. Using dendriplexes containing fluorescein-labeled non-targeting siRNA, we found that HM cells exhibited greater nanoparticle uptake than NM cells (Fig. 2S). Notably, DNM1 knockdown significantly reduced uptake in HM cells (Fig. 2T), suggesting that DNM1 could enhance targeted drug delivery to metastatic cells.

While some studies question the necessity of EMT for metastasis (Gerstberger et al., 2023), they often overlook the role of rapid endocytic mechanisms, which could provide a more physiologically relevant control over cancer cell motility (Aiello et al., 2018). Here, our bioinformatic analysis of multiple TCGA datasets has identified DNM1 as a novel mediator of EMT in ovarian cancer metastasis, facilitating N-cadherin endocytic recycling independent of TGF-β. The balance between E-cadherin and N-cadherin is crucial for EMT plasticity, with N-cadherin playing a vital role in promoting adhesive and pro-metastatic behaviors (Li et al., 2020; Mrozik et al., 2018). N-cadherin enhances the formation and invasion of multicellular aggregates, and its polarized recycling supports collective migration in plastic EMT cells (Mrozik et al., 2018). Targeting N-cadherin with monoclonal antibodies has shown promise in reducing metastasis (Mrozik et al., 2018). Interestingly, our findings indicate that post-translational glycosylation may influence N-cadherin recycling, offering valuable insights for more precise targeting strategies.

Elevated N-cadherin levels in ovarian cancer are associated with poorer outcomes, particularly in the mesenchymal molecular subtype, which correlates with severe complications and poor prognosis (Loret et al., 2019). Ovarian cancer is highly heterogeneous, with various histological subtypes affecting prognosis and treatment efficacy, and differences in the regulation of EMT among them. Studies have shown that non-serous subtype cell lines exhibit greater migratory and invasive capabilities compared to high-grade serous lines (Hallas-Potts et al., 2019). Histone deacetylase 9 (HDAC9) has been found to enhance EMT in serous subtypes by increasing nuclear forkhead box protein O1 (FOXO1), while it inhibits migration in non-serous cancers by suppressing β-catenin (Xu et al., 2022). The clinical data in this study focus on high-grade serous carcinoma, which represents about 75% of cases, therefore, the role of DNM1 in non-serous subtypes remains to be explored.

This study highlights two therapeutic implications: first, dysregulated endocytic recycling represents a promising target in plastic cells, potentially enhancing the effectiveness of current anti-cancer agents with minimal side effects (Banushi et al., 2023). Second, increased DNM1 expression enhances the uptake of therapeutic nanoparticles, suggesting that while EMT plasticity may lead to resistance to conventional therapies, it could increase responsiveness to nanoparticle-based interventions. Patient selection based on DNM1 expression could help identify those most likely to benefit from nanodrug formulations. In summary, our identification of the DNM1-N-cadherin axis provides new insights into EMT plasticity regulation, emphasizing the importance of investigating diverse mechanisms beyond classical transcription factors. Targeting EMT-associated endocytic recycling may yield more effective treatments for resistant metastatic cells in ovarian cancer and other aggressive tumors.

## Supplementary Material

pwaf019_suppl_Supplementary_Figures_S1-S4

pwaf019_suppl_Supplementary_Materials_and_Methods

## Footnotes

We thank Prof. Danny C. Y. Leung (Hong Kong University of Science and Technology) and Dr. Sophia S. N. Lam for assisting with the ATAC-seq data analysis.

This work was supported by funding from the RGC Senior Research Fellow Scheme SRFS2223-7S05 to A. S. T. Wong and from the “Laboratory for Synthetic Chemistry and Chemical Biology” under the Health@InnoHK Program launched by the Innovation and Technology Commission, Hong Kong SAR, China.

All animal studies conducted were ethically approved by the Committee on the Use of Live Animals in Teaching and Research at the University of Hong Kong, in accordance with the regulations outlined in the Animals (Control of Experiments) Ordinance of Hong Kong SAR, China. The use of patient specimens was approved by the Institutional Ethical Review Board for Research on the use of human subjects at the University of Hong Kong.

The authors declare no conflict of interest. All authors approve the publication of this manuscript. The data supporting this study are included in the manuscript and Supplementary Materials. The reported datasets are available from the corresponding author upon reasonable request. This paper does not report original code.

Y.C. and Z.G. conducted experiments and analyzed the data. Y.T. and J.Z. performed the bioinformatic analyses. Y.C., W.Z., and S.K.Y.T. prepared the manuscript. L.P. provided dendrimers. P.P.C.I. provided clinical samples. S.K.Y.T. and A.S.T.W. conceptualized the study, reviewed and edited the manuscript, and supervised the research.
