# Supplementary material for: Dynamin 1-mediated endocytic recycling of glycosylated N-cadherin sustains the plastic mesenchymal state to promote ovarian cancer metastasis
Source: Protein Cell. 2025 Apr 9;16(7):602–8. doi: 10.1093/procel/pwaf019 (PMC12275092; doi:10.1093/procel/pwaf019)
Supplement: pwaf019_suppl_Supplementary_Figures_S1-S4 [file pwaf019_suppl_supplementary_figures_s1-s4.pdf]

Figure S1

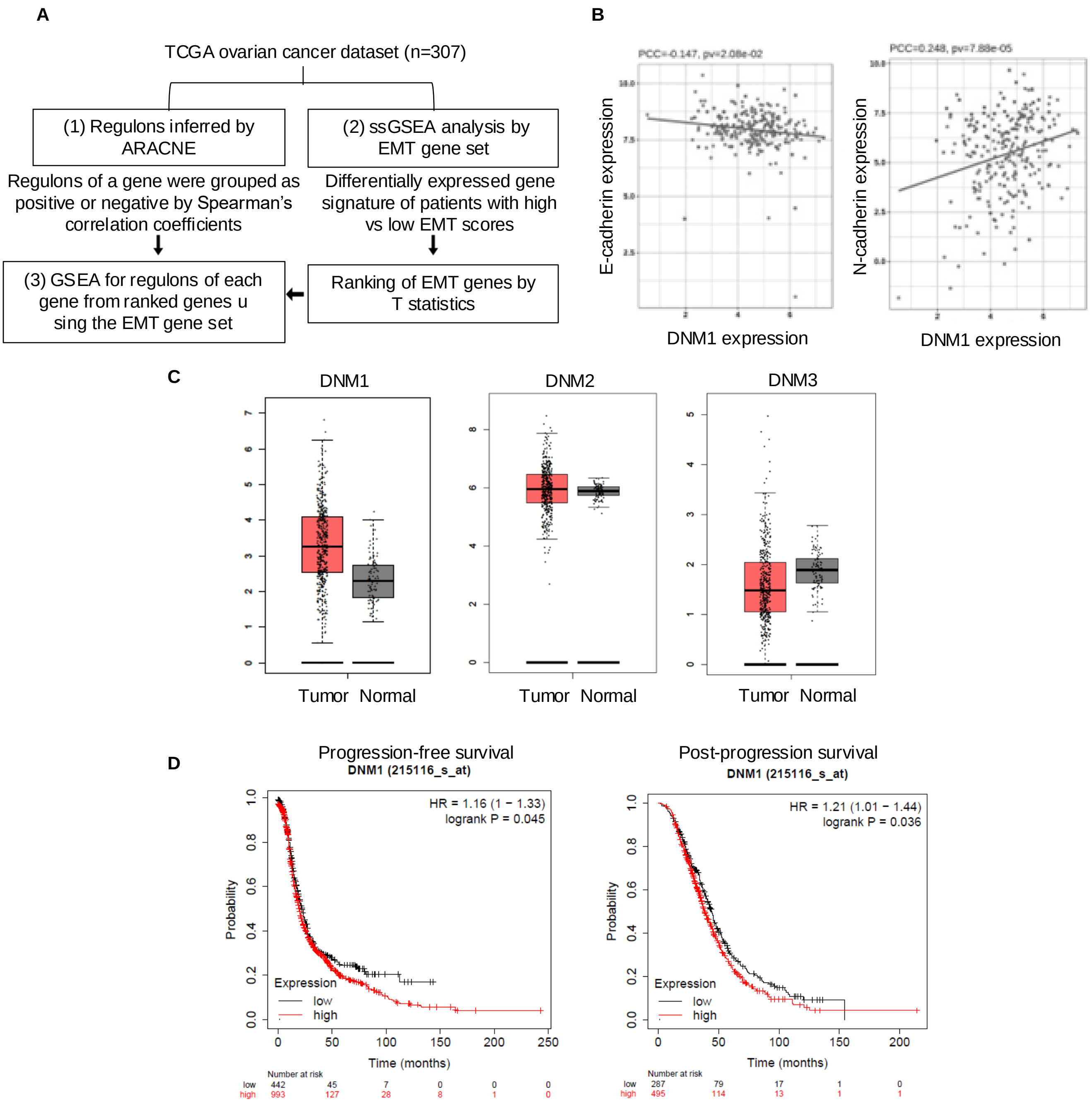

Figure S1. (A) A master regulator algorithm integrating TCGA ovarian cancer data and EMT signature was used to infer potential EMT markers. The basic workflow was shown: (1) Transcriptional targets (regulons) were inferred from the TCGA ovarian cancer gene expression profile using the Algorithm for the Reconstruction of Accurate Cellular Networks (ARACNE) with default parameters. Regulons were classified into positive (upregulated) and negative (downregulated) groups based on Spearman correlation coefficients between MR expression levels and each gene in its regulon; (2) A differential expression gene (DEG) analysis compared EMT-high tumors to EMT-low tumors. EMT scores were calculated using single sample gene set enrichment analysis (ssGSEA) from the HALLMARK M gene set to generate a ranked gene signature based on T statistics, and (3) The ranked gene list was analyzed with the GSEA method from the R "gage" package to assess the enrichment of regulons and their associated MRs based on FDR-adjusted P values. Only MRs with significantly enriched regulons (cut-off < 0.05) were selected for further validation. **(B)** Negative correlation between DNM1 and CDH1 (E-cadherin) expression (left panel) and positive correlation between DNM1 and CDH2 (N-cadherin) (right panel) expression were shown, evaluated by the Pearson Correlation Coefficient (PCC). **(C)** The mRNA expression levels of DNM1, DNM2, and DNM3 in ovarian cancer (n = 426) and normal tissues (n = 88) were analyzed using GEPIA (http://gepia.cancer-pku.cn). **(D)** Progression-free survival and post-progression survival of patients stratified by DNM1 expression level in ovarian cancer were analysed using Kaplan-Meier Plotter (https://kmplot.com/analysis/).

Figure S2

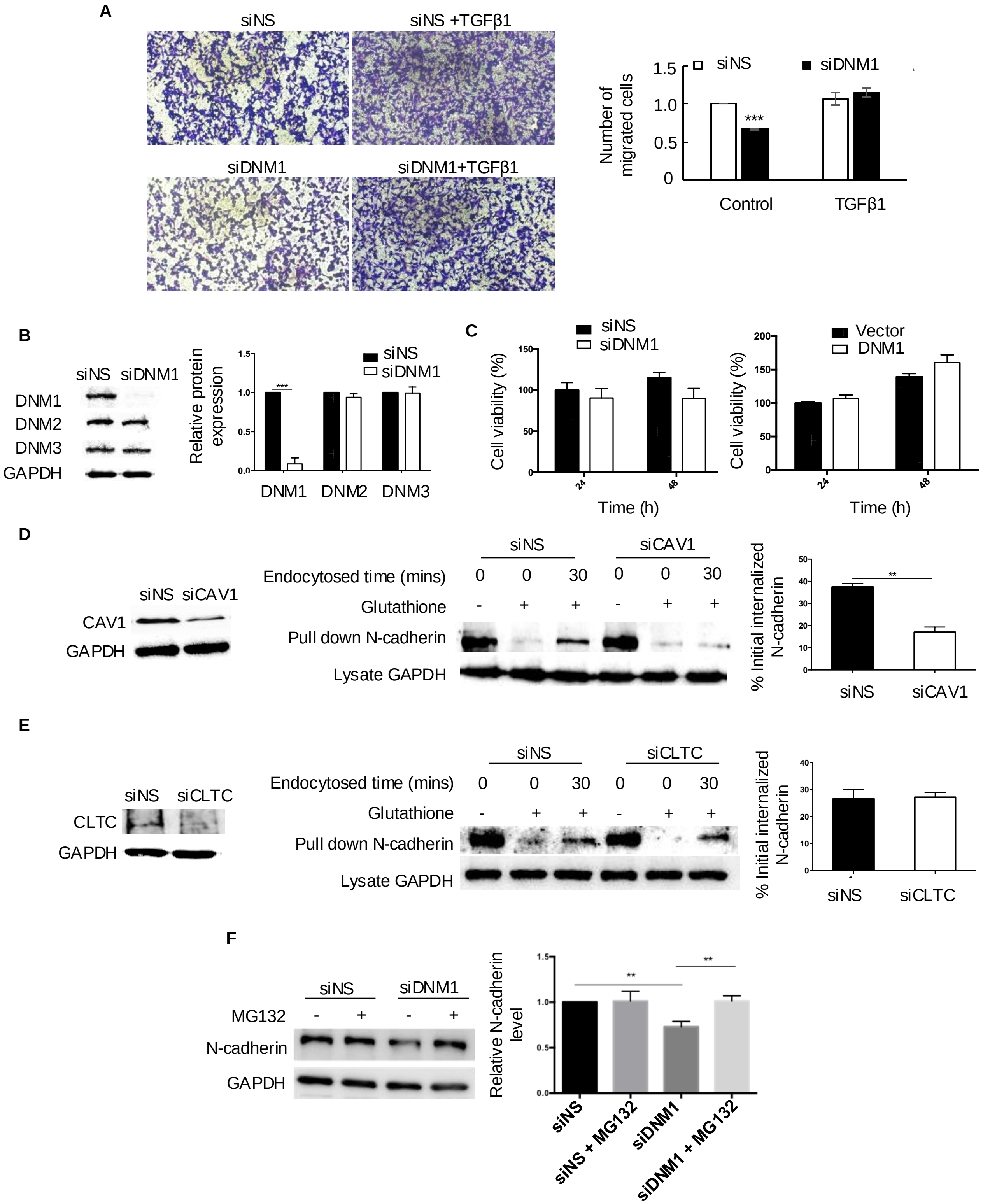

Figure S2. **(A)** HM cells transfected with non-specific (NS) or DNM1 siRNA were treated with or without TGF-β (10 ng/ml) as indicated for the migration assay. **(B)** Expression levels of DNM1, DNM2, and DNM3 were evaluated in HM cells transfected with NS or DNM1 siRNA. **(C)** MTT cell viability assays were conducted on HM cells treated with NS or DNM1 siRNA and NM cells transfected with either a control or DNM1 overexpression vector. **(D)** HM cells transfected with NS or CLTC siRNA were analyzed for clathrin expression by Western blot (left panel). The endocytosis of N-cadherin was compared between HM cells transfected with NS or caveolin-1 (CAV1) siRNA using a biotin-labeling assay followed by Western blot (right panel). **(E)** HM cells transfected with NS or CAV1 siRNA were assessed for caveolin expression by Western blot (left panel). HM cells transfected with NS or CLTC siRNA were analyzed using a biotin-labeling assay followed by Western blot (right panel). **(F)** HM cells transfected with DNM1 siRNA were treated with MG132 (50 µM) for 4 hours, and N-cadherin expression levels were detected by Western blot. Band intensities were quantified using ImageJ, with GAPDH as a loading control. Representative blots from three independent experiments are shown. *, $P < 0.05$; **, $P < 0.01$; ***, $P < 0.005$.

Figure S3

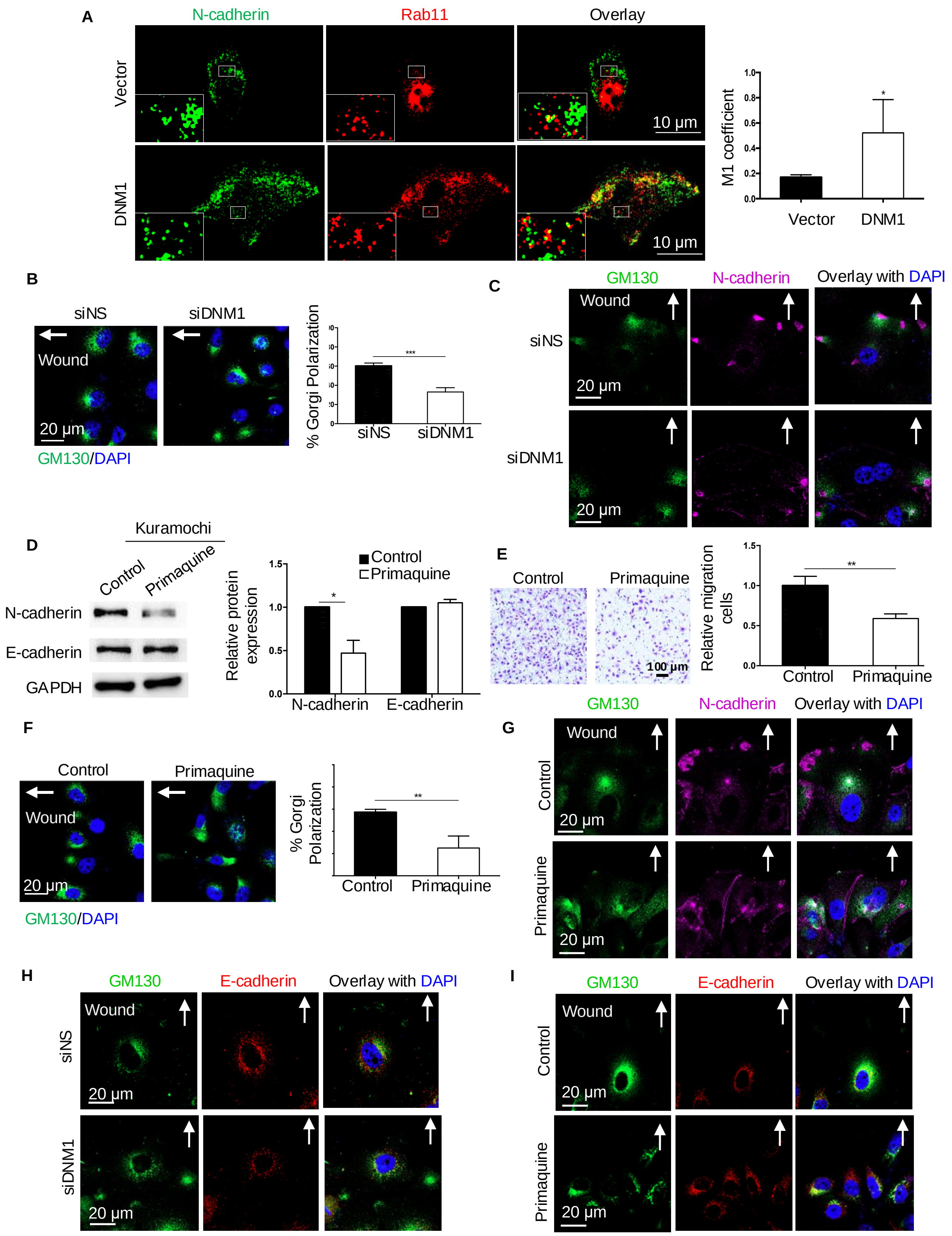
A
N-cadherin
Rab11
Overlay
Vector
DNM1
10 µm
10 µm
M1 coefficient
1.0
0.8
0.6
0.4
0.2
0.0
*
Vector
DNM1
B
siNS
siDNM1
Wound
20 µm
GM130/DAPI
% Gorgi Polarization
***
siNS
siDNM1
C
GM130
N-cadherin
Overlay with DAPI
siNS
siDNM1
Wound
20 µm
20 µm
D
Kuramochi
Control
Primaquine
N-cadherin
E-cadherin
GAPDH
Relative protein expression
1.5
1.0
0.5
0.0
Control
Primaquine
*
N-cadherin
E-cadherin
E
Control
Primaquine
100 µm
Relative migration cells
1.5
1.0
0.5
0.0
**
Control
Primaquine
F
Control
Primaquine
Wound
20 µm
GM130/DAPI
% Gorgi Polarization
**
Control
Primaquine
G
GM130
N-cadherin
Overlay with DAPI
Control
Primaquine
Wound
20 µm
20 µm
H
GM130
E-cadherin
Overlay with DAPI
siNS
siDNM1
Wound
20 µm
20 µm
I
GM130
E-cadherin
Overlay with DAPI
Control
Primaquine
Wound
20 µm
20 µm

Figure S3. **(A)** NM cells transfected with a control or DNM1 overexpression vector were stained for N-cadherin (green) and Rab11 (red), with colocalization of the two proteins observed through confocal imaging. Colocalization coefficient M1 was analyzed to describe the amounts of green pixels colocalizing with red pixels relative to the total green pixels. **(B)** Cell-free gaps were created in Kuramochi monolayers following transfection with NS or DNM1 siRNA. Golgi orientation was visualized by staining the Golgi marker GM130 (green), and the number of cells with correct polarity was counted. **(C)** Cells were co-stained with GM130 and N-cadherin (magenta) and observed by confocal imaging. **(D)** Kuramochi cells were treated with either vehicle control or primaquine (100 μM), a recycling inhibitor, and N-cadherin expression was assessed by Western blot. **(E)** Migration assays were performed on Kuramochi cells treated with either vehicle control or primaquine; migrated cells were stained with crystal violet and counted in five random fields of view. **(F)** Cell-free gaps were created in the HM monolayer treated with either vehicle control or primaquine. Golgi orientation was visualized by staining with the Golgi marker GM130 (green), and the number of cells with correct polarity was counted. **(G-I)** Kuramochi cells with the indicated treatment were co-stained with GM130 and **(G)** N-cadherin (magenta) or **(H and I)** E-cadherin (red) and observed by confocal imaging, with nuclei counterstained with DAPI (blue). Arrows indicate the expected Golgi orientation toward the wound. Representative data from three independent experiments are shown. *, $P < 0.05$; **, $P < 0.01$.

Figure S4

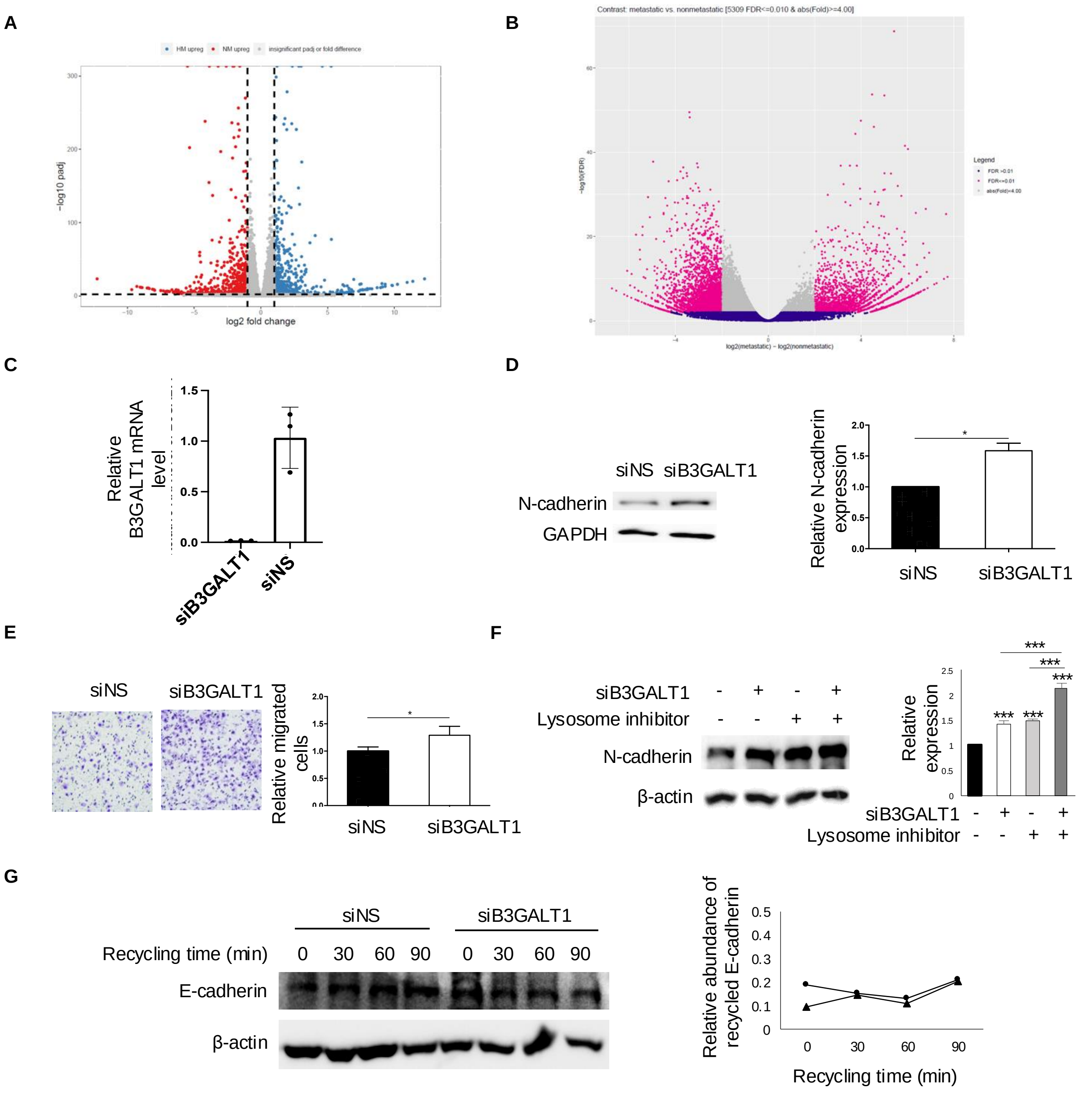

Figure S4. Volcano plots showing **(A)** differentially expressed genes (RNA-seq) and **(B)** differentially accessible peaks (ATAC-seq) between NM and HM. **(C)** HM cells transfected with NS or B3GALT1 siRNA were analyzed for B3GALT1 expression by qPCR. **(D)** N-cadherin expression was assessed by Western blot in Kuramochi cells transfected with NS or B3GALT1 siRNA, with GAPDH used as a loading control; band intensities were quantified using ImageJ. **(E)** Migration assays were conducted in Kuramochi cells transfected with NS or B3GALT1 siRNA. Migrated cells were stained with crystal violet and counted in five random fields of view. **(F)** NM cells were transfected with NS or B3GALT1 siRNA and treated with or without 50 mM NH_4Cl (lysosome inhibitor) for 4 hours. N-cadherin expression was detected by Western blot, with β-actin serving as a loading control. **(G)** NM cells were transfected with NS or B3GALT1 siRNA. After calcium chelation to induce cadherin internalization, cell surface proteins were allowed to recover for indicated time points. Cell surface proteins were biotinylated, and recycled E-cadherin was analyzed by Western blot. β-actin serves as a loading control. Representative data from three independent experiments are shown. *, $P < 0.05$; **, $P < 0.01$; ***, $P < 0.005$.
