# Supplementary material for: Dynamin 1-mediated endocytic recycling of glycosylated N-cadherin sustains the plastic mesenchymal state to promote ovarian cancer metastasis
Source: Protein Cell. 2025 Apr 9;16(7):602–8. doi: 10.1093/procel/pwaf019 (PMC12275092; doi:10.1093/procel/pwaf019)
Supplement: pwaf019_suppl_Supplementary_Materials_and_Methods [file pwaf019_suppl_supplementary_materials_and_methods.pdf]

Supplementary Materials

Materials and Methods

Master regulator prediction

Based on the ovarian cancer gene expression profile from The Cancer Genome Atlas Program (TCGA) dataset, transcriptional targets (or regulons) were inferred using the Algorithm for the Reconstruction of Accurate Cellular Networks (ARACNE) with default parameters. The regulons of a potential MR were then divided into positive (upregulated) and negative (downregulated) groups based on Spearman's correlation coefficients between the expression level of the MR and each gene in its regulon. To test EMT high tumors vs EMT low tumors, a differentially expressed gene (DEG) test was performed, and EMT scores were calculated using the single sample gene set enrichment analysis (ssGSEA) method from the HALLMARK EMT gene set to obtain a ranked differential gene signature (by T statistics). The ranked gene list was used as input for the GSEA method from the R "gage" package to evaluate the enrichment of the regulons, and their associated MRs based on the FDR-adjusted P values. Only MRs with significantly enriched associated regulons (cut-off < 0.05) were considered for further experiment validation.

Cell culture

The human ovarian cancer cell line HEYA8, OVSAHO, Kuramochi, and A2780 were cultured in RPMI 1640 medium (Sigma-Aldrich), while CaOV3, OVCAR3, and OVCA429 were grown in Medium 199:MCDB105 (Sigma-Aldrich) containing 5% fetal bovine serum (Hyclone) and 1% streptomycin and penicillin (Invitrogen). Cells were cultured in a humidified incubator with 5% CO2 at 37°C. The ovarian cancer cell lines were classified into four distinct categories: epithelial (OVSAHO, CaOV3, and OVCAR3), intermediate epithelial (OVCA429), intermediate mesenchymal (Kuramochi), and mesenchymal (HEYA8 and A2780). This classification was established by previous studies that analyzed and compared their transcriptomic EMT signatures, cell morphology, and/or expression levels of EMT markers (Huang et al., 2013; Tan et al., 2014; Terraneo et al., 2020).

Transfection

Transient transfection of plasmid DNAs (pEGFPN1 and pEGFPN1-DNM1 wild type) and short interfering RNA were performed using lipofectamine 2000 reagent (Invitrogen) for 48 h according to the manufacturer's instructions. The plasmid DNAs were purchased from Addgene, and the siRNAs were obtained from Dharmacon. Short

hairpin RNA (shRNA) stably expressing cells were generated using lentiviral plasmids carrying DNM1 shRNA (Sigma-Aldrich), followed by selection with 1 μg/ml puromycin (Calbiochem).

Western blot

The extracted proteins were separated by sodium dodecyl sulfate-polyacrylamide gel electrophoresis and transferred to the nitrocellulose membranes. The membranes were then blocked with 5% nonfat dry milk and incubated with primary antibodies. Corresponding HRP-conjugated secondary antibodies (Bio-rad) were incubated with the membranes at room temperature for 1 h. After washing 3 times, the membranes were detected using WESTERN LIGHTENING^{TM} Plus-ECL (Perkin Elmer) and imaged by the ChemiDoc MP System (Bio-rad). Antibodies against DNM1, N-cadherin, E-cadherin, vimentin, clathrin, and caveolin-1 were obtained from Cell Signaling Technology. The Dynamin 2 antibody was from Invitrogen, the Dynamin 3 antibody was from Abcam, and the GAPDH antibody was from Immunoway.

Real time polymerase chain reaction (qPCR)

Total RNA was isolated using Trizol reagent (Invitrogen), followed by reverse-transcription to cDNA using M-MLV reverse transcriptase (Invitrogen) according to the manufacturer's instructions. qPCR was performed using AceQ qPCR SYBR Green Master Mix (High ROX Premixed) Kit (Vazyme). Relative quantification was calculated by normalizing to the expression level of GAPDH using $2^{-\Delta\Delta Ct}$ method. The primer sequences used for qPCR are as follow:

GAPDH	Forward	5'-GGAGCGAGATCCCTCCAAAAT-3'
	Reverse	5'-GGCTGTTGTCATACTTCTCATGG-3'
DNM1	Forward	5'-CATTGGAGTGGTGAACCGGA-3'
	Reverse	5'-ACTTGAGACACGGTTCTCGG-3'
B3GALT1	Forward	5'-CGCTGGAGCCAACAGAGTTA-3'
	Reverse	5'-TCAGCATTCTTGCCCAGGAG-3'

Transwell migration assays

Cells were seeded into 24-well transwell inserts with serum-free medium and allowed to translocate toward the complete medium for 16 h. The migrated cells on the lower surface were fixed with ice-cold methanol and stained with 0.5% crystal violet. For the invasion assay, the inserts were pre-coated with Matrigel (BD Biosciences) and the same procedures were followed. Pictures of 5 random fields from each well were obtained using a microscope at × 10 magnification. The quantification was done by using ImageJ.

Surface biotinylation for endocytosis and recycling assays

Cells grown in 6-well plates were treated with 100 ug/ml leupeptin (Roche), 0.5 mM EGTA (Sigma), and 20 ug/ml cycloheximide (Calbiochem) for 30 min at 37°C to block lysosome activity and protein synthesis. Surface proteins were prelabeled with 0.5 mg/ml membrane-impermeable Sulfo-NHS-SS-Biotin (Thermo Scientific) on ice for 15 min. Unbound biotin was quenched with 50 mM NH_4Cl for 5 min at 4°C twice. Biotinylated cells were rinsed with fresh medium twice and then incubated at 37°C for 30 min. The cells were quickly chilled to 4°C, and the surface biotin was removed by incubating with 50 mM glutathione solution (75 mM NaCl, 75 mM NaOH, 50 mM reduced glutathione) twice for 15 min on ice. Free glutathione was quenched by 5 mg/ml iodoacetamide twice for 15 min on ice, followed by washing with PBS three times. The cells were then lysed by lysis buffer containing protease inhibitors. Biotinylated proteins were isolated by incubating with NeutrAvidin UltraLink Resin (Thermo Scientific) overnight at 4°C. The proteins were then separated and analyzed by Western blot. For recycling assays, cells were biotinylated and incubated at 37°C for 30 min and the remaining surface biotinylated proteins were removed by two rounds of glutathione treatment. Free glutathione was quenched by two rounds of iodoacetamide. The cells were rinsed with fresh medium and incubated at 37°C for 15 min, followed by two rounds of glutathione treatment and iodoacetamide treatments.

Immunofluorescence staining

For the colocalization assay, cells grown in 6-well plates were treated with 0.5 mM EGTA and 20 ug/ml cycloheximide for 30 min at 37°C to disrupt N-cadherin homophilic interactions and block protein synthesis. The cells were then incubated with antibody against the extracellular domain of N-cadherin (Invitrogen) for 1 h at 4°C. After the antibody incubation, cells were washed twice with fresh medium to remove unbound antibody, followed by incubation at 37°C for 15 min. After fixation with 4% paraformaldehyde, cells were blocked with 5% bovine serum albumin containing 0.5% Triton X-100 for 30 min prior to staining with Rab11 primary antibody overnight at 4°C. The cells were washed twice with PBS and further incubated with Cy3-conjuated anti-rabbit secondary antibodies and Cy5-conjuated anti-mouse secondary antibodies (Bio-rad) for 1 h. Cells were stained with Hoechst33342 to visualize the nuclei. The stained cells were mounted on glass slides with Vectashield mounting medium (Vecta Laboratories) and imaged with Zeiss ELYRA S1 super resolution microscope. For cell polarity analysis, cells were seeded on glass cover slips and cultured to full confluence in the presence of silicone inserts to create wound (Ibidi). The cells were further cultured for 6 h after insert removal, followed by fixation and staining with anti-N-

cadherin, anti-GM130 and Hoechst 33342, and imaged by a Carl Zeiss LSM 900 confocal microscope.

Sequencing

Total RNA was extracted from HM and NM cells with Trizol Reagent (Invitrogen). RNA-seq was performed at the Centre for PanorOmic Sciences (CPOS), the University of Hong Kong. For ATAC-seq, the Omni-ATAC-seq method was used with minor modifications (Corces, et al., 2017). Briefly, 50,000 viable cells were centrifuged at 500 g at 4°C for 5 min. The cell pellet was resuspended with 50 μL cold ATAC-Resuspension Buffer (RSB) (5 M NaCl, 1 M MgCl2, 10 mM Tris HCl pH 7.4 and protease inhibitor cocktail) containing 0.1% Igepal CA-630, 0.1% digitonin. After incubation on ice for 3 min, the sample was washed with 1 mL cold ATAC-RSB containing 0.1% tween-20, followed by centrifugation at 500 g for 10 min at 4°C. Tagmentation was performed by adding 50 μL transposase mix (16.9 μL PBS, 2.5 μL Tn5, 25 μL 2X TD buffer, 0.1 μL 5% digitonin, 0.5 μL 10% Tween-20 and 5 μL diH2O) and incubating in a thermocycler (1000 rpm) at 37°C for 30 min. Tagmented DNA was purified using the MinElute Kit. Library amplification was performed using indexed primers with 50 μL Kapa Hi Fi Hot Start PCR reaction for the first 5 cycles. 1 μL of amplified product was used to perform a library quantification with the KAPA library quantification kit to determine the optimum number of final amplification cycles. AMPure XP beads were used to select for fragments of 150 bp to 800 bp and the library size was determined by Fragment Analyzer. The obtained library was then sent to Novogene for library quality check, quantification, and sequencing.

Mice experiments

All mouse experiments were conducted in accordance with the guidelines of the University of Hong Kong Animal Care and Use Committee. Luciferase-expressing HM cells treated with NS or DNM1 shRNA (1×106) were injected intraperitoneally into 4-week-old female NOD/SCID mice (n=3/group). The mice were imaged twice a week for 22 days using the Xenogen IVIS system. The tumors on the omentum were harvested and metastatic nodules in the peritoneal cavity were counted. Formalin was used for tissue specimen fixation.

Immunohistochemistry (IHC)

FFPE sections were subjected to deparaffinization with xylene for 5 min three times and rehydration with graded ethanol (absolute ethanol, 95%, 70% and 50%, twice each). Antigen retrieval was performed using citrate buffer. After blocking, the slides were incubated with primary antibodies against PAX8, N-cadherin, and vimentin (Cell

Signaling Technology), and negative control at 4°C overnight. The slides were further processed using rabbit specific HRP/DAB (ABC) detection IHC kit according to the manufacturer's protocol (Abcam).

siRNA dendriplexes formation

AmDM (MW = 3838 g/mol, 16 amine end groups) was dissolved in distilled H2O and stocked at 500 μM. The cargo fluorescein-conjugated siRNA (Cell Signaling Technology) is a non-targeting siRNA without a specific silencing function. To prepare the siRNA dendriplexes, the dendrimer and siRNA were first diluted separately in OPTI-MEM (Invitrogen) and incubated at room temperature for 5 min. Dendrimer and siRNA (20 nM) were then mixed and incubated at room temperature for 20 min before treatment.

Statistics

Data were shown as mean ± SD and statistical analyses were conducted using student's t-test by GraphPad (San Diego, CA) for comparison between two groups. For comparisons involving three or more groups, one-way ANOVA was used for statistical analysis. $P < 0.05$ was considered statistically significant.

References

Huang, R.Y.; Wong, M.K.; Tan, T.Z.; Kuay, K. T.; Ng, A. H.; Chung, V. Y.; Chu, Y. S.; Matsumura, N.; Lai, H. C.; Lee, Y. F.; Sim, W. J.; Chai, C.; Pietschmann, E.; Mori, S.; Low, J. J.; Choolani, M.; Thiery, J. P. An EMT Spectrum Defines an Anoikis-Resistant and Spheroidogenic Intermediate Mesenchymal State That Is Sensitive to e-Cadherin Restoration by a Src-Kinase Inhibitor, Saracatinib (AZD0530). Cell Death Dis. 2013, 4, e915.

Tan, T.Z.; Miow, Q.H.; Miki, Y.; Noda, T.; Mori, S.; Huang, R. Y.; Thiery, J. P. Epithelial-Mesenchymal Transition Spectrum Quantification and Its Efficacy in Deciphering Survival and Drug Responses of Cancer Patients. EMBO Mol. Med. 2014, 6, 1279-1293.

Terraneo, N.; Jacob, F.; Peitzsch, C.; Dubrovska, A.; Krudewig, C.; Huang, Y. L.' Heinzelmann-Schwarz, V.; Schibli, R.; Béhé, M.; Grünberg, J. L1 Cell Adhesion Molecule Confers Radioresistance to Ovarian Cancer and Defines a New Cancer Stem Cell Population. Cancers 2020, 12, 217.
